# Late Spontaneous Migration of a Dorsal Column Stimulator Paddle Lead

**DOI:** 10.7759/cureus.740

**Published:** 2016-08-17

**Authors:** Chao Li, Michael A Galgano, David A Carter

**Affiliations:** 1 School of Osteopathic Medicine, Des Moines University; 2 Neurosurgery, SUNY Upstate Medical University

**Keywords:** lead migration, dorsal column stimulator, reflex sympathetic dystrophy, complex regional pain syndrome, late lead migration, paddle lead migration, late paddle lead migration, dorsal colum stimulation, spine surgery, cervical spine

## Abstract

The most frequently encountered complication of dorsal column stimulators is lead migration. The vast majority of these events are seen in the first few weeks to months. Late paddle lead migration is a very uncommon occurrence in this setting. We describe a case of a 51-year-old male with a history of reflex sympathetic dystrophy having undergone dorsal column stimulator insertion at the level of C1-C2. A good clinical benefit was appreciated in the postoperative period once the stimulator was turned on. Approximately six months postoperatively, the patient suddenly lost coverage. Radiographic imaging revealed that the lead had migrated caudally to the C3-C4 level. Subsequent revision surgery took place. This description highlights a common complication, but occurring outside the expected time frame after surgery.

## Introduction

Dorsal column stimulation has been utilized as a modality for pain relief in a variety of conditions ranging from complex regional pain syndrome (CRPS), failed back surgery syndrome (FBSS), and post-limb amputation pain [1]. The exact physiology of its ability to relieve pain is poorly understood. The proposed theory lies within concepts of the ‘gate-control’ theory of pain first introduced by Melzack and Wall in the 1960s [2]. Lead migration is the most common complication of dorsal column stimulator (DCS) insertion [3]. However, the vast majority of these complications occur less than six months post-implantation. After surgery, patients are instructed to refrain from flexing at the site of their surgery, due to the possibility of interspinous widening and subsequent migration of their lead. It is generally accepted that once an adequate epidural scar forms over the paddle lead, its chances of migration diminish. This is evidenced by the presence of scant literature describing late migrations of paddle leads after the six-month postoperative period.

## Case presentation

**Figure 1:**
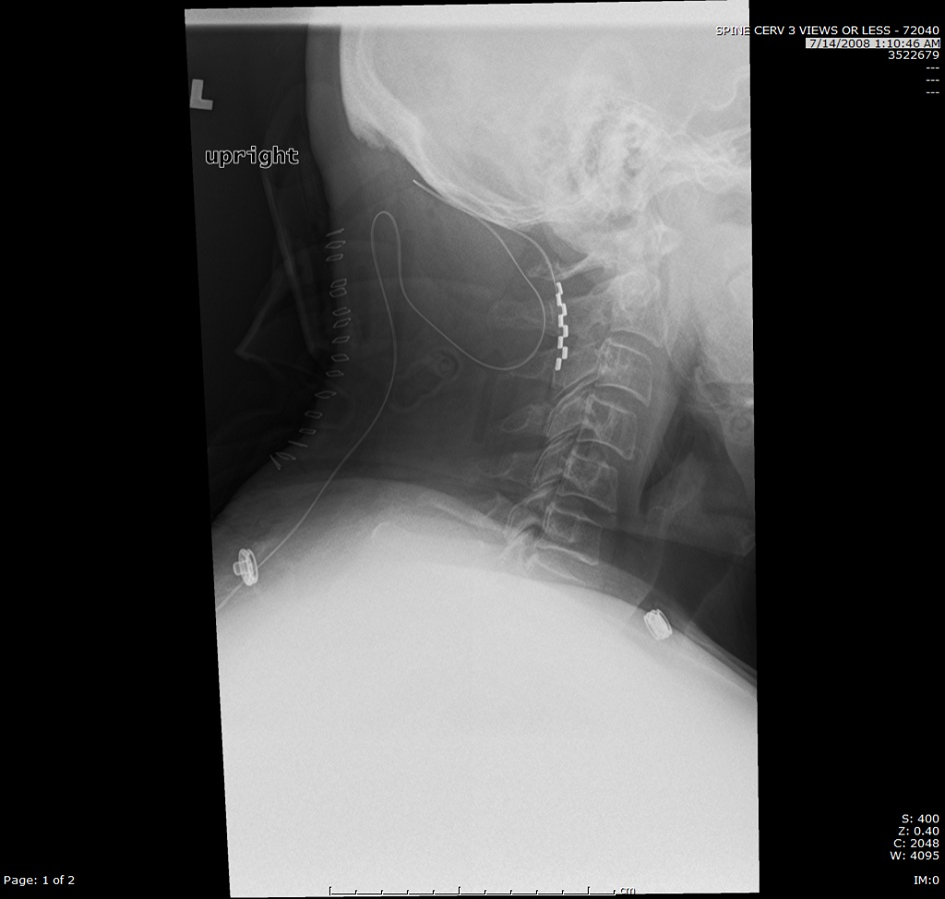
Lateral Cervical Spine X-Ray Dorsal column stimulator paddle lead at C1-C2

We report a case of a 51-year-old male with a history of chronic pain and complex regional pain syndrome. He had a dorsal column stimulator with paddle lead initially placed in 2006 after he developed chronic pain from a wrist fracture sustained from a work-related injury in 2004. He initially had some relief of the pain with the stimulator but later on developed problems abducting his left arm secondary to discomfort caused by the traversing leads. This interfered with his daily activities. In 2008, the patient underwent removal of his old hardware with partial C1-C2 laminectomies and placement of a new epidural dorsal column stimulator paddle lead at the level of C1-C2 and generator placement. Postoperative imaging revealed acceptable positioning of the paddle lead (Figure 1).

**Figure 2:**
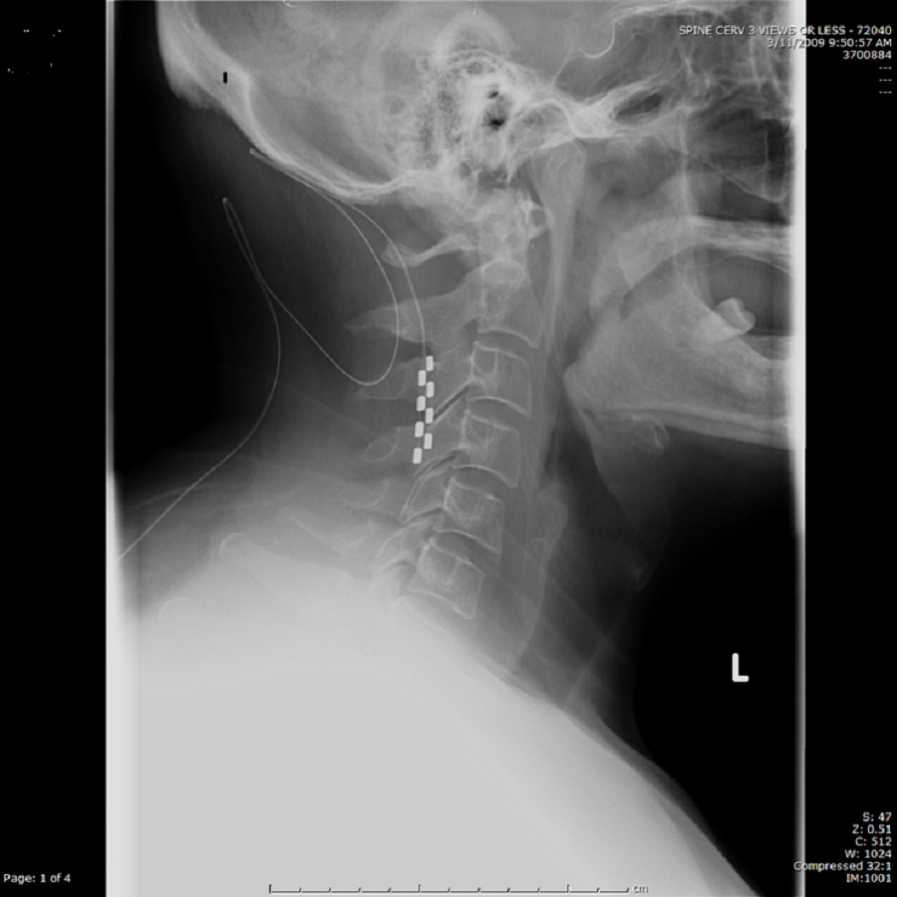
Lateral Cervical Spine X-Ray Caudal migration of dorsal column stimulator paddle lead to C3-C4

Excellent pain coverage was noted upon follow-up visits. However, six months after surgery, the patient spontaneously and suddenly lost pain coverage. He complained of a new “stinging” sensation in his right shoulder at the time. Although there was no specific inciting event leading up to the lead migration, the patient did have a  history of a 'tic disorder', which could have potentially played a role. Radiographic imaging revealed caudal migration of the lead from C1-C2 to C3-C4 (Figure 2)

**Figure 3:**
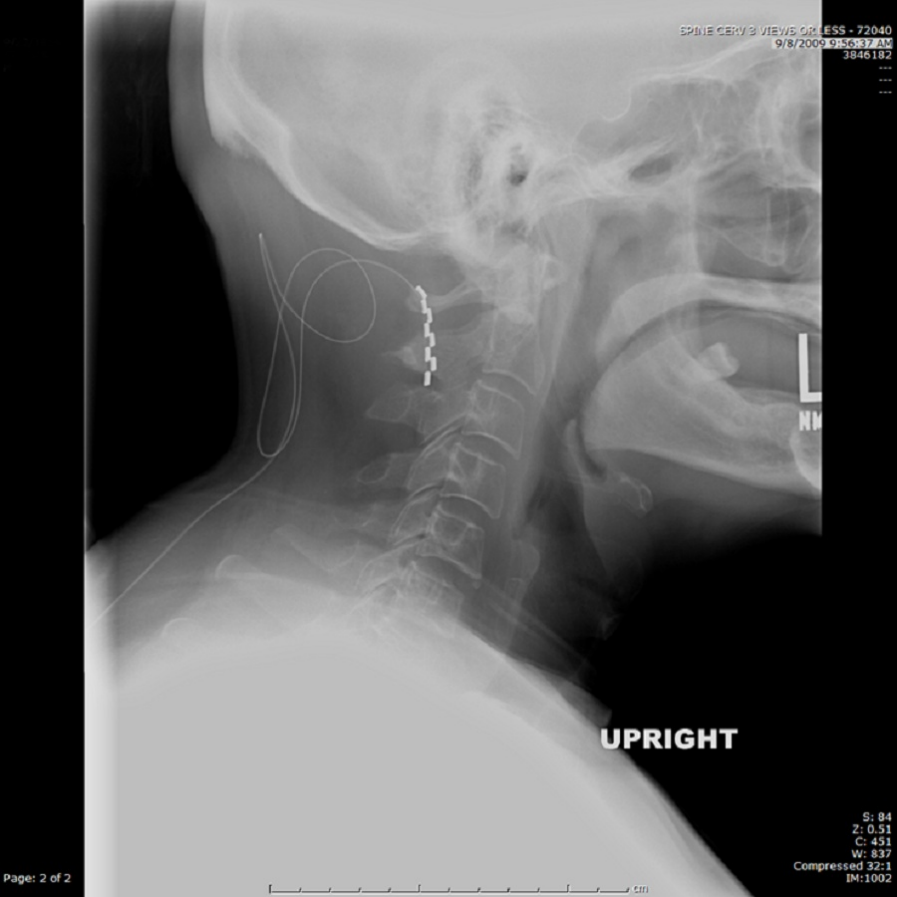
Lateral Cervical Spine X-Ray One-year follow-up image revealing dorsal column stimulator paddle lead at C1-C2

Subsequent revision surgery was undertaken. The lead was found to be in proper position at one-year follow-up (Figure 3).

The patient agreed to participate and was explained the nature and objectives of this study, and informed consent was formally obtained. No reference to the patient's identity was made at any stage during data analysis or in the report.

## Discussion

Dorsal column stimulation has been shown to provide pain relief for conditions such as CRPS, FBSS, and post-limb amputation pain. Literature suggests that there is between 50% and 70% successful pain relief based on improvements in pain severity scores, the overall functionality of the patient, and decreased dependence on pain medications [4-9]. Overall, lead migration is a common complication occurring between 0.0% and 13.5% of the time in cases [3, 10-14]. Paddle electrode lead migration rate was reported to be about 4.8% and generally believed to be less common than percutaneous cylinder lead placement [14]. This typically occurs less than six months after implantation with an average of about 125 days [1]. The latest reported paddle electrode migration was reported about 2.2 years after surgery [14].

One factor that may play a role in paddle lead migration is the size of the laminectomy defect created for the insertion. If too small of a laminectomy is done, a paddle lead may cause a mass effect in an already stenotic spinal canal, potentially leading to neurological sequelae. Adequate bone removal must be undertaken in order for the spinal canal to accommodate the lead. If overly zealous removal of the posterior osseous elements nevertheless takes place, the paddle lead may be prone to migration. In an effort to avoid lead migration with a rather extensive laminectomy, it has been suggested that the lead should be sutured to the dura in a partial thickness fashion [15]. This technique should be done with great caution due to the potential for an iatrogenic cerebrospinal fluid leak or incorporation of the neural elements into the needle. A technique that we utilize entails placing a suture through the interspinous ligament, prior to wrapping it around the leads, in an effort to tether the unit to the spinous process.

Patient-related factors, such as movement disorders, have the potential to play a role in lead migration as well. 

## Conclusions

Migration of both percutaneous and paddle DCS leads is an event complicating greater than 10% of all insertions. Patients are instructed to refrain from flexing at their laminectomy insertion level for a few weeks postoperatively to avoid interspinous widening with subsequent migration of the lead. It is felt that epidural scar formation over the lead will eventually hold it in place permanently. Prior to significant scar formation, however, the lead is vulnerable to shifting out of its placed position. Our case occurred outside of the expected time frame for such a complication, suggesting that some individuals may take longer to form a significant scar, or that some individuals are prone to migration despite adequate scar formation. The exact etiology of the late lead migration in this case described is unknown, especially since it occurred spontaneously without any antecedent history of trauma or significant neck flexion. Due to the relatively high rates of paddle lead migration in general, refined anchoring methods should be devised in an effort to decrease the incidence of this complication.
